# The Prostaglandin E2 Receptor EP4 Regulates Obesity-Related Inflammation and Insulin Sensitivity

**DOI:** 10.1371/journal.pone.0136304

**Published:** 2015-08-26

**Authors:** Mika Yasui, Yukinori Tamura, Manabu Minami, Sei Higuchi, Risako Fujikawa, Taichi Ikedo, Manabu Nagata, Hidenori Arai, Toshinori Murayama, Masayuki Yokode

**Affiliations:** 1 Department of Clinical Innovative Medicine, Kyoto University Graduate School of Medicine, Kyoto, Kyoto, Japan; 2 Department of Physiology and Regenerative Medicine, Kinki University Faculty of Medicine, Osakasayama, Osaka, Japan; 3 Department of Neurosurgery, Kyoto University Graduate School of Medicine, Kyoto, Kyoto, Japan; 4 National Center for Geriatrics and Gerontology, Obu, Aichi, Japan; Graduate School of Medicine, Osaka University, JAPAN

## Abstract

With increasing body weight, macrophages accumulate in adipose tissue. There, activated macrophages secrete numerous proinflammatory cytokines and chemokines, giving rise to chronic inflammation and insulin resistance. Prostaglandin E_2_ suppresses macrophage activation via EP4; however, the role of EP4 signaling in insulin resistance and type 2 diabetes mellitus remains unknown. In this study, we treated *db/db* mice with an EP4-selective agonist, ONO-AE1-329, for 4 weeks to explore the role of EP4 signaling in obesity-related inflammation *in vivo*. Administration of the EP4 agonist did not affect body weight gain or food intake; however, in the EP4 agonist–treated group, glucose tolerance and insulin resistance were significantly improved over that of the vehicle–treated group. Additionally, administration of the EP4 agonist inhibited the accumulation of F4/80-positive macrophages and the formation of crown-like structures in white adipose tissue, and the adipocytes were significantly smaller. The treatment of the EP4 agonist increased the number of anti-inflammatory M2 macrophages, and in the stromal vascular fraction of white adipose tissue, which includes macrophages, it markedly decreased the levels of proinflammatory cytokines and chemokines. Further, EP4 activation increased the expression of adiponectin and peroxidase proliferator–activated receptors in white adipose tissue. Next, we examined *in vitro* M1/M2 polarization assay to investigate the impact of EP4 signaling on determining the functional phenotypes of macrophages. Treatment with EP4 agonist enhanced M2 polarization in wild-type peritoneal macrophages, whereas EP4-deficient macrophages were less susceptible to M2 polarization. Notably, antagonizing peroxidase proliferator–activated receptor δ activity suppressed EP4 signaling-mediated shift toward M2 macrophage polarization. Thus, our results demonstrate that EP4 signaling plays a critical role in obesity-related adipose tissue inflammation and insulin resistance by regulating macrophage recruitment and polarization. The activation of EP4 signaling holds promise for treating obesity and type 2 diabetes mellitus.

## Introduction

Obesity predisposes to several metabolic diseases such as insulin resistance, type 2 diabetes mellitus (T2DM), and arteriosclerosis. Developing novel approaches for the prevention and treatment of T2DM is a matter of great importance.

Excess calorie intake contributes to increased body weight, which is associated with larger adipocytes, preadipocyte differentiation, and abnormal adipokine secretion [1]. Hypertrophic adipocytes secrete monocyte chemotactic protein-1 (MCP-1), which promotes macrophage infiltration into obese adipose tissue, thus inducing chronic, low-grade inflammation [2]. White adipose tissue (WAT) is an important site for obesity-related chronic inflammation where adipose tissue macrophages (ATMs) produce proinflammatory cytokines and chemokines, such as tumor necrosis factor alpha (TNFα) and MCP-1 [3]. The activation of inflammatory signaling can trigger whole-body insulin resistance by directly influencing insulin signaling [4–6].

There are two major phenotypes for macrophages, M1 (classically activated) and M2 (alternatively activated) [7]. M1 macrophages produce proinflammatory cytokines and are induced by Th1 cell–derived interferon (IFN) γ and lipopolysaccharide (LPS). By contrast, M2 macrophages reduce inflammatory responses by producing anti-inflammatory factors, such as interleukin (IL)-10 and transforming growth factor (TGF)-β [7], and mainly reside in lean adipose tissue. When mice on a high-fat diet become obese, M1 macrophages accumulate in the adipose tissue, resulting in a shift toward M1 polarity, suggesting that the M1/M2 polarization of ATMs plays a key role in insulin resistance and T2DM [8].

Prostanoids, comprised of prostaglandin and thromboxane, are bioactive compounds synthesized in response to various stimuli and are crucial for maintaining tissue homeostasis and inflammation [9]. Prostaglandin E_2_ (PGE_2_) is one of the major prostanoids generated by the metabolism of arachidonic acid by cyclooxygenase (COX) and PGE synthases [10]. It has four G protein–coupled receptors: EP1 through EP4 [11]. PGE_2_ suppresses the production of proinflammatory cytokines and chemokines via EP4 in LPS–treated human and murine macrophages [12,13]. In fact, EP4 activation suppresses chronic inflammation *in vivo* by mitigating macrophage activation during afflictions such as inflammatory bowel disease [14], ischemia-reperfusion injury [15], atherosclerosis [16], allograft rejection after cardiac transplantation [17], and abdominal aortic aneurysm [18]. In addition, EP4 signaling suppresses adipocyte differentiation [19] and protects against the diabetogenic toxicity of streptozotocin in mice [20]. PGE_2_ mediates, at least partially, the biological effects of adiponectin (a crucial anti-inflammatory and anti-atherosclerotic molecule including suppression of adipocyte differentiation [21] and inhibition of ischemia-reperfusion injury [22].

These findings imply important roles for EP4 signaling *in vivo* in obesity-related chronic inflammation and in the subsequent increase of insulin resistance. In this study, we pharmacologically activated the EP4 receptor in *db/db* mice to investigate the pathophysiological role of EP4 signaling in obesity and T2DM.

## Results

### Treatment with an EP4-selective agonist significantly improves glucose tolerance and insulin resistance in obese mice

**Fig 1:**
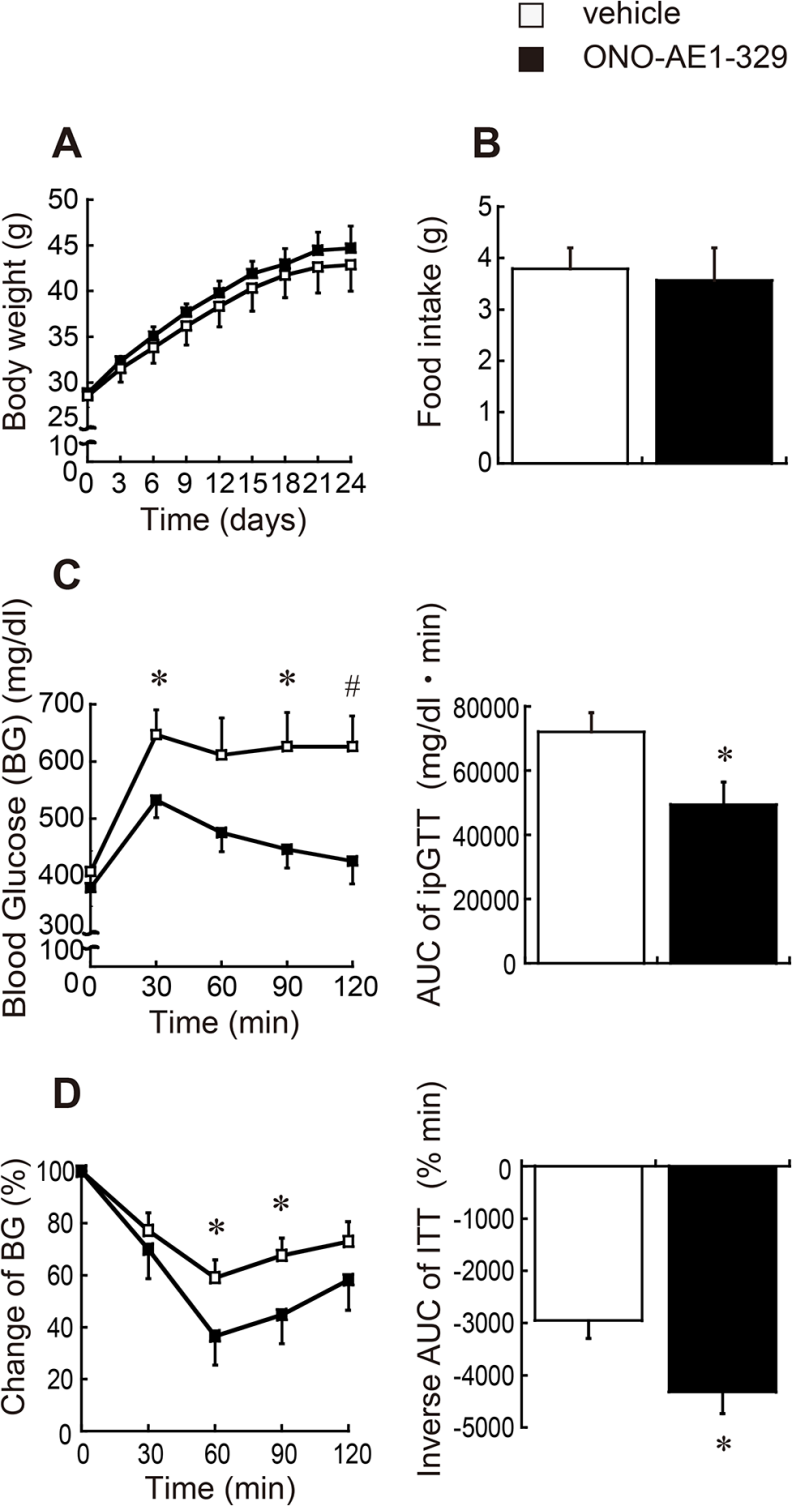
Effects of EP4 activation on glucose tolerance and insulin resistance. Seven-week-old male *db/db* mice subcutaneously received either the EP4-selective agonist ONO-AE1-329 (0.3 mg/kg) or vehicle twice a day for 4 weeks. (A) Change in body weight during the study period (n = 8 each). (B) Amount of food intake (n = 8 each). (C) Results of glucose tolerance test: left, time-course of blood glucose levels; right, area under curve (AUC) (n = 7–8 each). (D) Results of insulin tolerance test: left, time-course changes of blood glucose levels; right, the inverse AUC (n = 13 each). All values are mean ± SEM. * p<0.05; ♯ p<0.01 vs. vehicle.

To determine the role of EP4 signaling in obesity-related inflammation *in vivo*, we administered the EP4-selective agonist ONO-AE1-329 or vehicle for 4 weeks to *db/db* mice. The dosage of the EP4 agonist was determined in accordance with previous reports using animal models of inflammation [23,24]. ONO-AE1-329 treatment did not affect body weight gain (Fig 1A) or food intake (Fig 1B). There was no significant difference in the plasma levels of total cholesterol, triglyceride, or hemoglobin A1C between the two groups (Table 1). Additionally, liver weight and epididymal WAT did not differ significantly (Table 1). Despite similar metabolic characteristics, treatment with the EP4 agonist markedly improved glucose tolerance (Fig 1C). In addition, the insulin tolerance test indicated that the EP4 agonist increased insulin sensitivity (Fig 1D). Thus, EP4 activation ameliorated obesity-induced abnormal glucose tolerance and insulin resistance.

**Table 1:** Effects of EP4 agonist on metabolic parameters.

Measurement	Vehicle	ONO-AE1-329	p-value
T-Cho (mg/dl)	117.35 ± 21.71	115.51 ± 12.16	N.S.
TG (mg/dl)	141.27 ± 37.18	153.06 ± 33.89	N.S.
HbA1c (%)	6.28 ± 0.42	6.05 ± 0.22	N.S.
Liver (g)	2.49 ± 0.25	2.43 ± 0.17	N.S.
Epididymal WAT (g)	2.35 ± 0.09	2.30 ± 0.15	N.S.

Values are mean ± SEM (n = 3–8 each). T-Cho, total cholesterol; TG, triglyceride; HbA1c, Hemoglobin A1c; WAT, white adipose tissue.

### EP4 signaling inhibits macrophage accumulation in crown-like structures and inflammatory activation in obese adipose tissues

**Fig 2:**
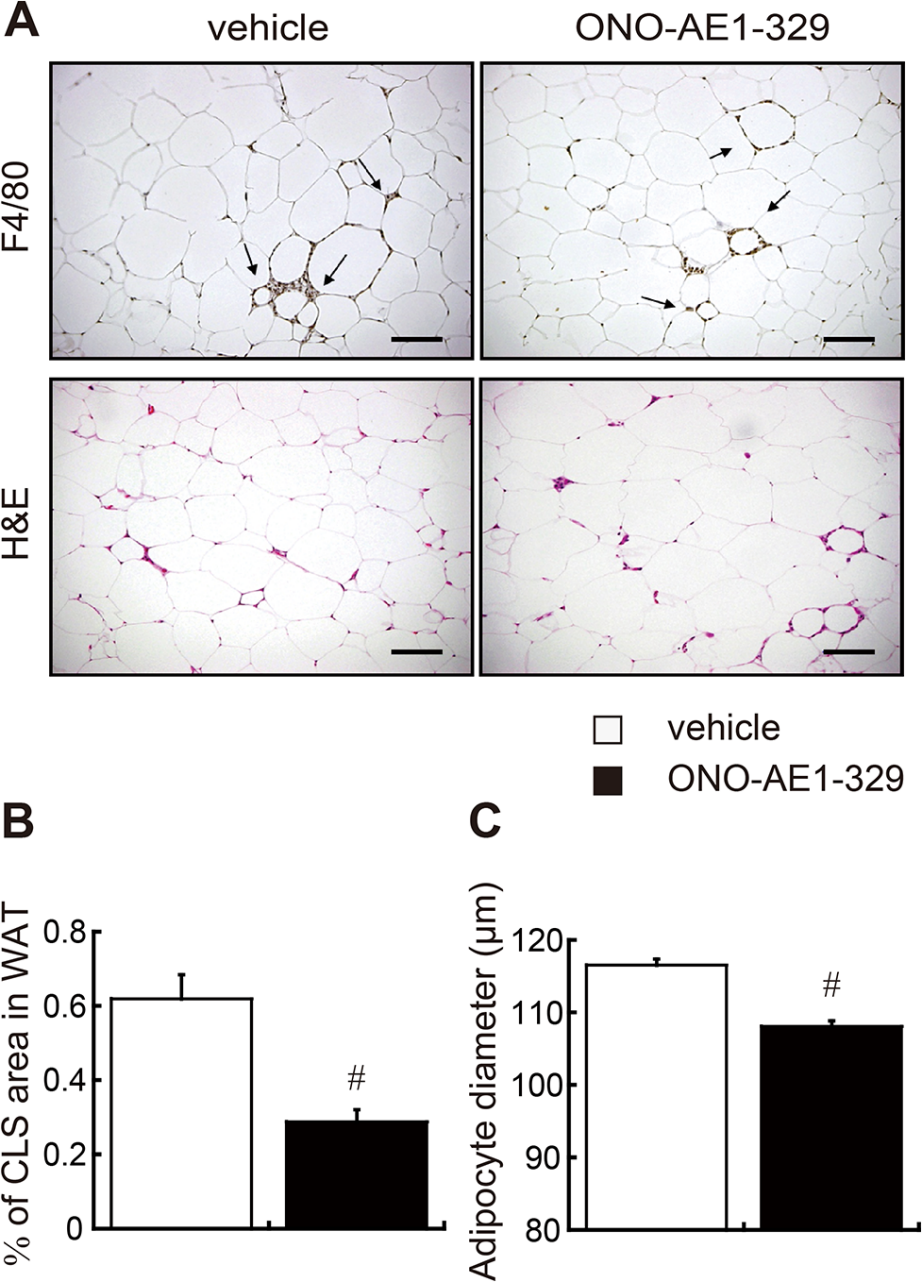
Adipose tissue morphology. (A) Representative images of epididymal adipose tissue stained with anti-F4/80 antibody (upper panel; the arrows indicate F4/80-positive cells) and H&E (lower panel) in *db/db* mice administered EP4 agonist (right column) or vehicle (left column). Scale bars: 100 μm. (B) Percent of F4/80^+^ CLS areas in mouse epididymal adipose tissue (n = 7 each). (C) Quantification of adipocyte size (n = 7 each). All values are mean ± SEM. ♯ p<0.01 vs. vehicle. CLS, crown-like structures.

Accumulation of macrophages in adipose tissues elicits inflammation, which in turn, causes local and systemic insulin resistance and T2DM [2,25]. Because several lines of evidence indicate that EP4 signaling in macrophages has anti-inflammatory effects, we next investigated the impact of EP4 activation on inflammatory burden in obese adipose tissue. Immunohistochemistry revealed that the accumulation of F4/80-positive macrophages in crown-like structures (CLSs) within epididymal WAT was markedly decreased in the EP4 agonist–treated group (Fig 2A and 2B). Accordingly, the sizes of adipocytes in the EP4 agonist–treated group were significantly smaller compared to that of the vehicle–treated group (Fig 2A and 2C). Though there was no significant difference in the mRNA expression of *Mcp-1* in WAT (S1A Fig), there was a trend towards decreased secretion of MCP-1 protein in the EP4 agonist–treated mice (p = 0.054) (S1B Fig).

**Fig 3:**
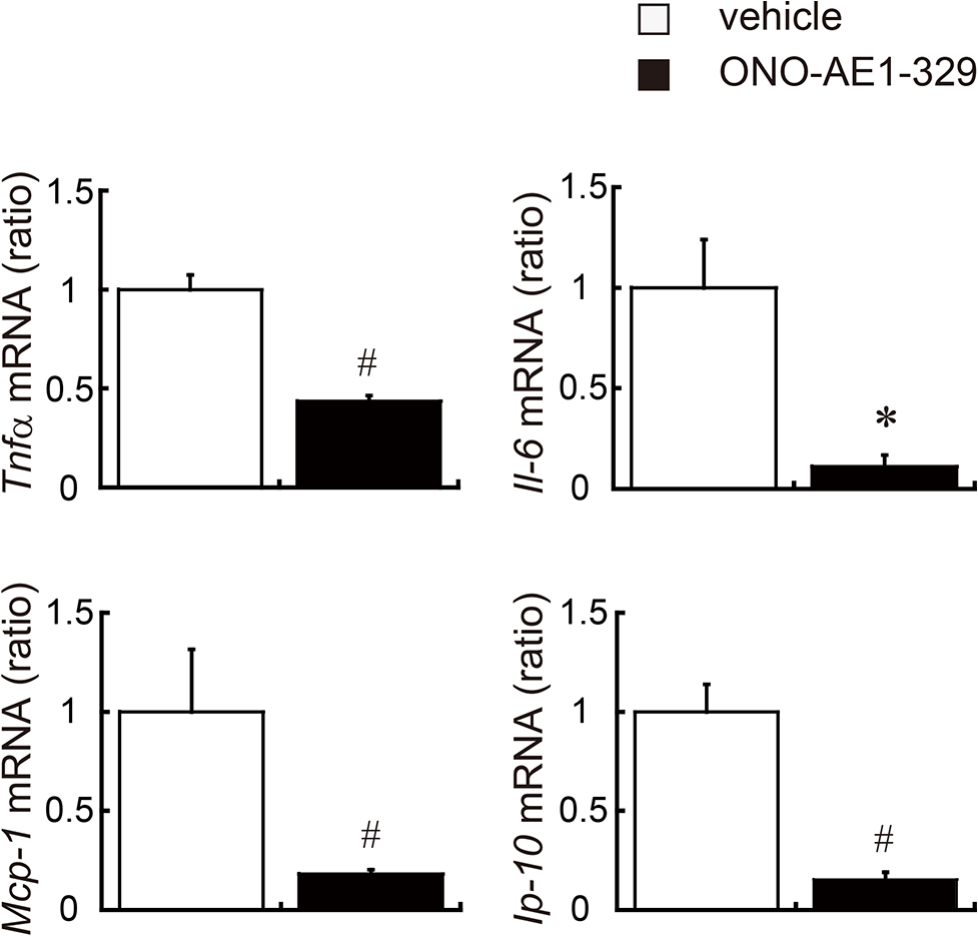
Cytokine and chemokine gene expression in SVF. Relative expression of *Tnfα*, *Il-6*, *Mcp-1*, and *Ip-10* mRNA in SVF isolated from *db/db* mice administered EP4 agonist (black bar) or vehicle (white bar). All values are mean ± SEM (n = 3 each). * p<0.05; ♯ p<0.01 vs. vehicle. SVF, stromal vascular fraction; Tnfα, tumor necrosis factor α; Il-6, interleukin-6; Mcp-1, monocyte chemotactic protein-1; Ip-10, interferon gamma–induced protein 10.

To investigate the effect of EP4 activation on ATM activation, we isolated stromal vascular fraction (SVF) from epididymal WAT, which contains inflammatory cells including ATMs, and then measured the mRNA levels of proinflammatory cytokines and chemokines. The EP4 agonist–treated group had decreased expression of a number of genes encoding proinflammatory cytokines and chemokines such as *Tnfα*, *Il-6*, *Mcp-1*, and interferon gamma–induced protein 10 (*Ip-10*) (Fig 3). These results indicate that activation of EP4 signaling has potent anti-inflammatory effects in ATMs.

### EP4 signaling enhances the polarization of adipose tissue macrophages toward the M2 phenotype

**Fig 4:**
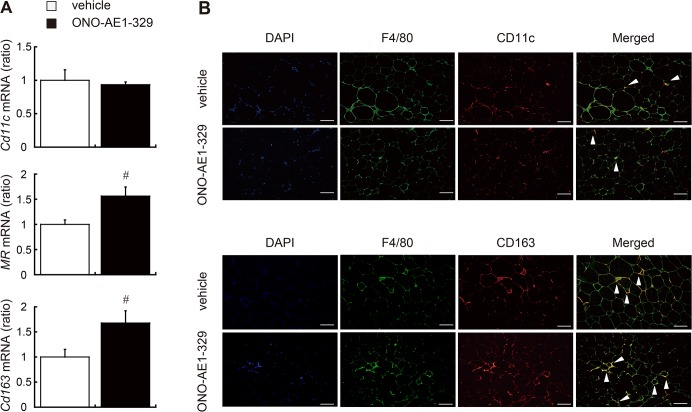
Administration of EP4 agonist alters adipose tissue macrophage polarization. (A) Relative expression of marker genes for M1 (*Cd11c*) and M2 (*MR*, *Cd163*) macrophages in epididymal fat tissues from *db/db* mice administered EP4 agonist (black bar) or vehicle (white bar). All values are mean ± SEM (n = 4–5 each). ♯ p<0.01 vs. vehicle. (B) Epididymal adipose tissues of EP4 agonist–or vehicle–treated *db/db* mice were double stained with anti-F4/80 (green), and anti-CD11c (red, upper panel) or anti-CD163 (red, lower panel) antibodies. Arrows indicate double-positive cells. Scale bar: 100 μm. MR, Mannose Receptor.

ATMs are comprised of two distinct subsets: the classically activated, proinflammatory M1 macrophages and the alternatively activated, anti-inflammatory M2 macrophages. The total number of infiltrated macrophages and the M1/M2 balance of those macrophages determine the features of chronic inflammatory diseases such as obesity and T2DM [8,26]. To evaluate the polarization of ATMs, we performed quantitative PCR on total mRNA from epididymal WAT. The expression of genes encoding the M2 markers mannose receptor (*MR*) and *Cd163* was increased in the EP4 agonist–treated group, whereas the gene encoding the M1 marker *Cd11c* was not significantly different between the two groups (Fig 4A). Peroxidase proliferator–activated receptor (PPAR) δ and PPARγ are key factors in polarizing macrophages to M2 status [26–29]. In our study, the expression of these genes in WAT was significantly increased by consecutive administration of EP4 agonist (S2 Fig). Accordingly, immunofluorescence of epididymal WAT demonstrated that F4/80^+^ CD163^+^ M2 macrophages, not F4/80^+^ CD11c^+^ M1 macrophages, were more abundant in the EP4 agonist–treated group (Fig 4B).

### Impact of EP4 signaling on the balance of adipokine secretion

**Fig 5:**
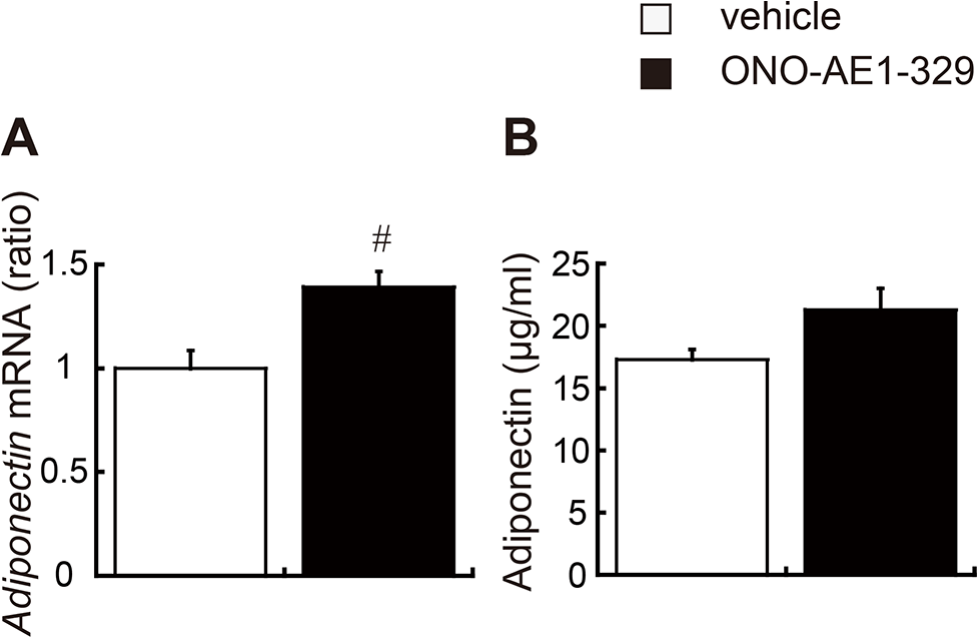
Impact of EP4 activation on adiponectin expression. Relative adiponectin mRNA levels in epididymal fat tissue (A) and plasma adiponectin concentrations (B) were measured in EP4 agonist–(black bar) or vehicle–treated (white bar) *db/db* mice. All values are mean ± SEM (n = 6 each). ♯ p<0.01 vs. vehicle.

Adipokines, including adiponectin, are produced and secreted from fat cells in adipose tissues and regulate systemic insulin sensitivity [30]. The nuclear receptors PPARα and PPARγ regulate the expression of adiponectin [31,32]. Adiponectin, in turn, suppresses macrophage infiltration into adipose tissue and promotes alternative M2 macrophage activation [33,34]. In the EP4 agonist–treated group, the expression levels of *PPARα* and *PPARγ* in WAT were significantly increased, along with production of adiponectin (S2 Fig and Fig 5A). Consequently, though not statistically significant (p = 0.079), the plasma levels of adiponectin exhibited a possible trend toward significance in the EP4 agonist–treated group (Fig 5B), suggesting that polarization of ATMs toward the anti-inflammatory M2 phenotype balanced adipokine secretion and improved insulin sensitivity in obese mice.

### EP4 signaling promotes M2 macrophage polarization in isolated murine macrophages

**Fig 6:**
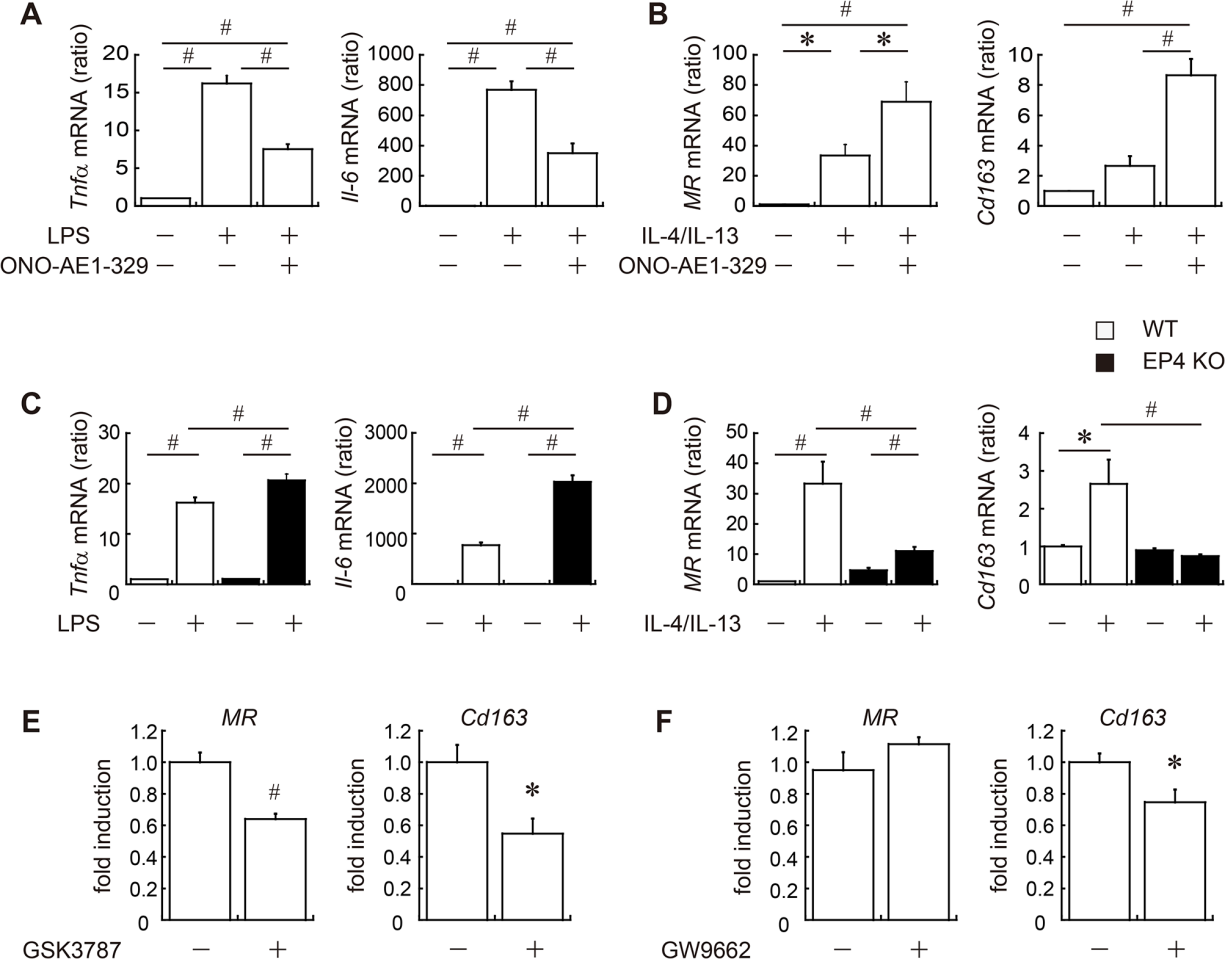
EP4 signaling is important in macrophage polarization. *In vitro* M1/M2 polarization assays were performed using peritoneal macrophages freshly isolated from 12 to 15-week-old male WT (white bar) or EP4-deficient mice (black bar). To determine M1 or M2 polarization, cells were incubated with 1 μg/ml of LPS (A, C), or with 20 ng/ml of IL-4 and IL-13 (B, D), as well as with 1 μM of EP4 agonist or vehicle. Eight hours later, marker gene expression for M1 (*Tnfα* and *Il-6*) (A, C) or M2 (*MR* and *Cd163*) (B, D) polarity was measured. To clarify the role of PPARs in EP4-dependent M2 polarization, peritoneal macrophages were pretreated with GSK3787 (PPARδ antagonist) (E), GW9662 (PPARγ antagonist) (F) or vehicle prior to the administration of IL-4/IL-13 and the EP4 agonist. Results are expressed as the fold induction of M2 genes compared with unantagonized, vehicle-treated cells. All values are mean ± SEM (n = 6 each). * p<0.05; ♯ p<0.01. LPS, lipopolysaccharide.

To investigate whether EP4 signaling directly influences macrophage polarization, we freshly isolated peritoneal macrophages from WT mice and performed *in vitro* M1/M2 polarization assays as previously described [35]. The expression of marker genes for M1 and M2 macrophages was assayed by quantitative PCR. In WT macrophages, LPS stimulation induced the mRNA expression of M1 genes, such as *Tnfα* and *Il-6*, whereas adding the EP4 agonist to the LPS mixture decreased the mRNA expression of those genes (Fig 6A). On the other hand, co-stimulation with IL-4 and IL-13 enabled peritoneal macrophages to polarize to M2 status (Fig 6B). Notably, treatment with the EP4 agonist with IL-4 and IL-13 further increased the mRNA expression of M2 genes, including *MR* and *Cd163* (Fig 6B).

To investigate the impact of EP4 deletion on macrophage polarization, we collected peritoneal macrophages from EP4 knockout mice and estimated M1/M2 polarization. EP4 deficiency resulted in increased susceptibility to M1 and less susceptibility to M2 macrophage polarization (Fig 6C and 6D), suggesting that EP4 signaling may act directly on macrophage polarization. These results indicate that the activation of EP4 signaling mitigates chronic inflammation in adipose tissue by regulating ATM activation and polarization.

Next, we examined the downstream of EP4 signaling that enhanced M2 polarization. It is well known that the activation of PPARs is one of the keys in polarizing macrophages to M2 status [26–29]. When we added GSK3787, PPARδ selective antagonist, prior to IL-4/IL-13 in *in vitro* M1/M2 polarization assays, the EP4-dependent increase of *Cd163* or *MR* gene expression markedly inhibited (Fig 6E). On the other hand, pretreatment of GW9662, PPARγ antagonist, suppressed the enhanced expression of *Cd163* but not *MR* by EP4 agonist (Fig 6F). These results suggest that PPARs, especially PPARδ, might coordinate EP4 signaling in macrophage polarization toward an M2-like phenotype.

## Discussion

Prostaglandin E_2_ (PGE_2_) has been shown to exert energy and metabolic homeostasis: for instance, PGE_2_ regulates lipolysis and increases leptin release in primary culture of rodent and human adipose tissues [36–38], while EP3-deficient mice displayed increased feeding and body weight despite elevated plasma leptin levels [39]. In addition, a previous report demonstrated that intracerebroventricular administration of PGE_2_ or EP4 agonist decreased food intake in fasted mice [40]. In this study, however, administration of EP4 selective agonist did not affect body weight gain, food intake or blood lipid profile. It may be partially because that in our study, we used leptin receptor-deficient *db/db* mice fed with normal chow *ad libitum*, and administered EP4 agonist peripherally. Besides, in WAT, other EP receptors including EP3 express and activate intracellular signal transduction pathways differing from EP4.

EP4 activation is pivotally involved in ameliorating chronic inflammatory diseases [14–16,41]. Macrophages, known to have abundant EP4 expression [12], accumulate in hypertrophied adipose tissues and play crucial roles in the pathogenesis of insulin resistance and T2DM by promoting inflammation. Therefore, we examined the impact of EP4 signaling on obesity-related adipose tissue inflammation *in vivo* using an animal model of obesity and T2DM.

Obesity is generally caused by the combination of excess calorie intake, insufficient physical activity, and genetic predisposition. Obese adipose tissue is characterized by hypertrophied adipocytes, increased production of proinflammatory cytokines and chemokines, and infiltration by immune cells, including macrophages [42]. Though there was no significant difference in the weight of epididymal adipose tissue in our study, the adipocytes were less hypertrophied in the EP4 agonist–treated group. Hypertrophied adipocytes secrete MCP-1, leading to the recruitment of circulating monocytes and subsequent infiltration by macrophages into adipose tissues. Infiltrated ATMs, in turn, secrete MCP-1 and other proinflammatory cytokines and chemokines; consequently a number of ATMs accumulate in adipose tissues, causing chronic inflammation in WAT [2,8,25]. PGE_2_-EP4 signaling suppresses MCP-1 expression in a variety of inflammatory settings [43,44]. Our data demonstrated that consecutive administration of EP4 receptor–agonist into obese mice significantly decreased MCP-1 production in SVF, a fraction containing macrophages but not adipocytes, suggesting that EP4 signaling mainly affects ATMs and inhibits the deleterious consequences of local chemokine production and macrophage infiltration.

Obesity causes a shift in macrophage polarity from the anti-inflammatory M2 to the pro-inflammatory M1 state, facilitating insulin resistance [8]. Using mice with mutations in key proteins of the inflammatory process, it has been demonstrated that an increase in M2 ATMs ameliorates obesity-related impaired glucose metabolism. In one example, mice lacking Tribbles homolog 1 (*Trib1*, which encodes an adaptor protein involved in proteasome-mediated protein degradation) in hematopoietic cells develop glucose intolerance and insulin resistance on a high-fat diet. They have a severely reduced number of M2 ATMs, but no change in M1 ATMs [45]. Hematopoietic deletion of COX-1, a major PGE_2_-producing enzyme, was also associated with metabolic disorders and decreased counts of M2 ATMs in diet-induced obese mice, with M1 counts remaining the same [46]. How M2 ATMs contribute to the amelioration of glucose tolerance and insulin resistance is not fully understood; however, a number of previous reports indicated that M2 macrophages reside in adipose tissues have crucial roles in maintaining normal glucose homeostasis [3,47,48]. Polarized M2 ATMs secrete anti-inflammatory cytokines such as IL-10, improving insulin-stimulated glucose uptake in TNF*α*–treated 3T3-L1 adipocytes [8]. In addition to secreting cytokine or regulating adipokine production, some of M2 ATMs can store large amount of iron: loss of these alternatively activated macrophages may cause abnormal accumulation of iron in adipocytes and enhance iron-induced lipid peroxidation and oxidative stress, which consequently deteriorate adipocyte insulin sensitivity [49]. Besides, M2 ATMs are capable of producing catecholamines such as norepinephrine, activating β3 adrenergic receptor on adipocytes: β3 adrenergic receptor signaling in WAT is pivotally involved in metabolic homeostasis through regulating lipolysis and mitochondrial functions [50,51].

Accordingly, we observed that the activation of EP4 signaling increased the number of M2 macrophages and led to a phonotypic switch toward M2 status in obese adipose tissue. Notably, in our study, EP4 signaling pivotally participated in M1/M2 differentiation *in vitro*, and in EP4 agonist–treated mice, the gene expression of *PPARδ* and *PPARγ* in WAT were significantly elevated. PPARδ is induced in response to adipocyte-derived Th2 cytokines, such as IL-4 and IL-13, and facilitates adipocyte differentiation, as well as the recruitment of M2 macrophages into adipose tissue and the liver [27,28]. PPARγ is necessary for the alternative activation of ATMs [26,29]. Thus, our data suggest that EP4 activation regulates macrophage accumulation in obese adipose tissue, as well as differentiation and polarization toward M2 phenotypes, resulting in the suppression of adipose tissue inflammation.

Adipose tissues secrete various adipokines, and the plasma adipokine levels can be an indicator of systemic inflammation and insulin resistance. Adiponectin is one of the major adipokines, exhibiting anti-diabetic, anti-atherosclerotic, and anti-inflammatory properties [52]. The activation of PGE_2_-EP4 signaling is crucial for the protective function of adiponectin [22]. In addition, adiponectin promotes the alternative activation of macrophages and polarization toward the anti-inflammatory M2 phenotype [34,53]. Our study indicates that consecutive EP4 activation induces local adiponectin production in WAT, which in turn, might contribute to the phenotypic shift of ATMs into M2 status and suppression of adipose tissue inflammation. In contrast to enhanced adiponectin mRNA levels in WAT, we observed no statistically significant change in the plasma levels of total adiponectin in EP4 agonist–treated group; yet some previous works demonstrated that insulin sensitivity does not always correlate with the plasma total adiponectin levels, because adiponectin circulates in the blood in multimeric forms with different metabolic activities [54,55].

Defining the precise mechanisms by which EP4 activation enhances M2 polarization is beyond the scope of the current study; however, as discussed above, successive treatment of obese mice with EP4-selective agonist enhances *PPARγ*, *PPARδ*, and adiponectin expression, which may explain the polarity shift of accumulated macrophages, at least partially. EP4 is coupled to Gs, which stimulates adenylate cyclase, and thus, increases intracellular cAMP levels, consequently activating protein kinase A and cAMP response element binding protein (CREB) [56]. In differentiated 3T3-L1 adipocytes, adiponectin expression is mediated through cAMP–CREB–PPARγ pathways [57], but it remains controversial whether PGE_2_ or EP4 signaling directly acts on the expression of these genes via a cAMP-dependent pathway. Unlike other EP receptors coupled with Gs, EP4 has a long cytoplasmic tail, where the receptor interacts with a novel protein designated EP4 receptor–associated protein (EPRAP) [12]. Because EPRAP exerts an anti-inflammatory effect in LPS-stimulated macrophages *in vitro* [12,13], EPRAP may also contribute to the suppression of obesity-related inflammation.

In this study, we focused on the impact of EP4 signaling in adipose tissues on macrophage activation and polarization. PGE_2_ regulates the profibrotic and proinflammatory activation of pancreatic stellate cells via EP4 and protects β-cells from apoptosis [58,59]. Other than adipose tissues, macrophages also infiltrate into the liver and the skeletal muscle in obese subjects, and the polarity appears to be shifted towards M1 status, which has a strong association with dysfunction of these organs and with insulin resistance [27,28,60]. Clarifying the roles of EP4 signaling in macrophage activation and phenotypic switching within these organs could be important for understanding the novel pathophysiological mechanisms of insulin resistance and T2DM.

In summary, administration of an EP4-selective agonist improved insulin sensitivity and glucose tolerance in obese mice. Treatment with EP4 agonist inhibited the accumulation of macrophages and decreased CLS formation, consequently attenuating the expression of proinflammatory cytokines and chemokines, and enhancing adiponectin production in white adipose tissue. EP4 activation promoted ATM polarization toward the M2 phenotype in obese mice; accordingly, our *in vitro* experiments verified that EP4 signaling played a crucial role in the differentiation and polarization of murine peritoneal macrophages. Further investigation to clarify the detailed molecular mechanisms underlying the effects of EP4 signaling on macrophage differentiation and polarization in other insulin-target organs will provide new insights into the pathogenesis, as well as novel therapeutic targets, of obesity-related insulin resistance and T2DM.

## Materials and Methods

### Ethics statement

All animal care and experiments were conducted following the guidelines for the Japan’s Act on Welfare and Management of Animals (Act No. 105 of October 1, 1973). These studies were approved by the Institutional Animal Care and Use Committees (IACUC)/ethics committee of Kyoto University (Permit Number: MedKyo15183). All sections of this report are based on the ARRIVE Guidelines for reporting animal research [61]. A completed ARRIVAL guidelines checklist is included in S1 Checklist. All surgery was performed when mice were anesthetized by 40 mg/kg of pentobarbital sodium (Kyoritsu Seiyaku, Tokyo, Japan); blood and tissue collection were performed as terminal procedures under anesthesia as described above, and all efforts were made to minimize suffering.

### Animals

Five-week-old male *db/db* mice were obtained from Oriental BioService (Kyoto, Japan). Homozygotic EP4-deficient mice and WT mice with the same genetic background were obtained by crossing mice heterozygous for the *PTGER4* mutation and were verified as previously described [62]. We used 26 *db/db* mice housed in groups of 2. Other varieties of mice had 6 mice per group and were housed in groups of 6. Mice were maintained in a specific pathogen-free facility (12 h light/dark cycles) and were fed normal chow *ad libitum* unless otherwise indicated.

### Treatment of db/db mice with EP4 receptor–selective agonist

ONO-AE1-329 (Ono Pharmaceutical, Osaka, Japan), an EP4-selective agonist [14,63], was suspended in vehicle (0.3% ethanol and 0.1% Tween 80 in PBS), and 0.3 mg/kg of the reagent or vehicle was administrated subcutaneously to 7-week-old *db/db* mice twice a day for 4 weeks. We allocated animals into 2 groups as the vehicle–treated group and the ONO-AE1-329–treated group with 13 mice based on body weight (vehicle: 38.93 ± 0.62, ONO-AE1-329: 39.78 ± 0.52; mean ± SE, no significant difference) at the start of the experiment in order to minimize the effect of subjective bias.

### Glucose and insulin tolerance test

After an overnight fast, we administered 1.5 g/kg of D(+)-glucose (Wako Pure Chemical Industries, Osaka, Japan) and 0.75 U/kg of Humulin R (Eli Lilly Japan, Kobe, Japan) intraperitoneally to perform the glucose tolerance test (ipGTT) and the insulin tolerance test (ITT), respectively.

### Plasma variables

Plasma levels of blood glucose (BG), total cholesterol (T-Cho), triglyceride (TG), hemoglobin A1c (HbA1c), and adiponectin were measured using the Glucose CII Test Wako (Wako), the Total Cholesterol E-Test Wako (Wako), the Triglyceride E-Test Wako (Wako), the Glycohemoglobin A1c kit (Sanwa Kagaku Kenkyusho, Nagoya, Japan), and the Adiponectin ELISA kit (Otsuka Pharmaceutical, Tokyo, Japan), respectively.

### Histology

Epididymal adipose tissue was harvested and fixed overnight with 4% paraformaldehyde (Wako), embedded in paraffin, and sectioned. For H&E staining, we stained the rehydrated sections using Mayer’s hematoxylin solution (Wako). For immunohistochemistry, the rehydrated sections were blocked with Protein Block Serum-Free (Dako, Glostrup, Denmark) and the macrophages were stained using rat monoclonal anti-F4/80 antibody (Abcam, Cambridge, MA, USA; clone CI:A3-1, 1:100; AB_1140040) and visualized by coloring with diaminobenzidine (DAB; Vector Laboratories, Burlingame, CA, USA). For immunofluorescence staining, blocked sections were incubated with a combination of rat anti-F4/80 antibody (Abcam; clone CI:A3-1, 1:100; AB_1140040) and either rabbit polyclonal anti-CD11c antibody (Santa Cruz Biotechnology, Dallas, TX, USA; clone M-50, 1:100; AB_2129774) or rabbit polyclonal anti-CD163 antibody (Santa Cruz Biotechnology; clone M-96, 1:100; AB_2074556). After washing, the sections were incubated with Alexa Fluor 488–conjugated donkey anti-rat antibody (Invitrogen, Carlsbad, CA, USA) or Alexa Fluor 594–conjugated donkey anti-rabbit antibody (Invitrogen). Non-immune rat IgG (Vector Laboratories) and rabbit IgG (Santa Cruz Biotechnology) served as negative controls for each experiment.

### Quantitative PCR

Total RNA was extracted from adipose tissue, stromal vascular fraction (SVF), and peritoneal macrophages using the RNeasy Lipid Tissue Mini kit (Qiagen, Valencia, CA, USA), RNeasy Micro kit (Qiagen), and RNeasy Mini kit (Qiagen), respectively. RNA was reverse-transcribed by High-Capacity cDNA Reverse Transcription Kits (Applied Biosystems, Forster City, CA, USA). Samples were processed on the 7300 Real-Time PCR System (Applied Biosystems) using the Power SYBR Green PCR Master Mix (Applied Biosystems). All experiments were performed in duplicate, and results were normalized to *β-actin* expression level. The sequences of the sense and antisense primers used for amplification are as follows; *β-actin*: 5'-CCTGAGCGCAAGTACTCTGTGT-3’, 5'-GCTGATCCACATCTGCTGGAA-3’; *Tnfα*: 5'-CATCTTCTCAAAATTCGAGTGACAA-3’, 5'-TGGGAGTAGACAAGGTACAACCC-3’; *Il-6*: 5'-TAGTCCTTCCTACCCCAATTTCC-3’, 5'-TTGGTCCTTAGCCACTCCTTC-3’; *Mcp-1*: 5'-GCTGGAGCATCCACGTGTT-3’, 5'-ATCTTGCTGGTGAATGTGTAGCA-3’; *IP-10*: 5’-GCCGTCATTTTCTGCCTCAT-3’, 5’-GCTTCCCTATGGCCCTCATT-3’; *Cd11c*: 5'-CTGGATAGCCTTTCTTCTGCTG-3’, 5'-GCACACTGTGTCCGAACTC-3’; *MR*: 5'-GCTGAATCCCAGAAATTCCGC-3’, 5'-ATCACAGGCATACAGGGTGAC-3’; *Cd163*: 5'-GGGTCATTCAGAGGCACACTG-3’, 5'-CTGGCTGTCCTGTCAAGGCT-3’; *Adiponectin*: 5'- GATGGCAGAGATGGCACTCC-3’, 5'-CTTGCCAGTGCTGCCGTCAT-3’; *PPARδ*: 5'-AGATGGTGGCAGAGCTATGACC-3’, 5'-TCTCCTCCTGTGGCTGTTCC-3’; *PPARγ*: 5'-CTCCAAGAATACCAAAGTGCGA-3’, 5'-GCCTGATGCTTTATCCCCACA-3’; *PPARα*: 5'- ATGCCAGTACTGCCGTTTTC-3’, 5'-CCGAATCTTTCAGGTCGTGT-3’.

### Isolation of stromal vascular fraction (SVF)

Epididymal adipose tissues were minced and digested in 0.2% collagenase (Wako) in PBS for 2 h at 37°C. The digested tissues were passed through a 100 μm nylon mesh filter (BD Biosciences, San Jose, CA, USA) to remove the floating adipocytes, and the filtrates were spun for 5 min at 1,200 rpm. The pellet was incubated with Lysing Buffer (BD Biosciences) for 2 min to remove the red blood cells.

### In vitro M1/M2 polarization assay

Peritoneal macrophages were extracted from the peritoneal lavage of 12 to 15-week-old male EP4-deficient mice or WT mice with the same genetic background. The *in vitro* M1/M2 polarization assay was performed as previously described [35]. Briefly, together with 1 μM of ONO-AE1-329 or vehicle, peritoneal macrophages were treated with 1 μg/ml of LPS (*Escherichia coli* O55: B5, Calbiochem, La Jolla, CA, USA) or 20 ng/ml of IL-4 (R&D Systems, Minneapolis, MN, USA) and IL-13 (R&D Systems) for 8 h. Meanwhile, to explore the roles of PPARs in EP4-dependent M2 polarization, 0.1 μM of GSK3787 (Tocris Bioscience, Bristol, UK), selective antagonist for PPARδ, or 1 μM of GW9662 (Cayman Chemical, Ann Arbor, MI, USA), selective antagonist for PPARγ, was added into the medium 2h before IL-4/IL-13 and the EP4 agonist treatment.

### Cytometric bead assay

Epididymal WAT was homogenized in RIPA buffer (Wako) with Protease inhibitor cocktail (Roche Diagnostics, Indianapolis, IN, USA). MCP-1 concentration in the supernatants of epididymal WAT homogenates was measured using the BD CBA assay (BD Biosciences).

### Statistical analysis

Results are presented as the mean ± SEM. All data were analyzed with Kaleida Graph software (version4.1J; Synergy Software, Reading, PA, USA). Differences between two value sets were determined using the unpaired, two-tailed Student’s *t*-test. A one-way ANOVA followed by Turkey-Kramer analysis was used for testing the differences among three or more groups. Significance was defined as a p-value less than 0.05.

## Supporting Information

S1 Checklist Completed ARRIVE Guidelines Checklist for reporting animal data in this manuscript. (PDF)

S1 Fig Impact of EP4 activation on MCP-1 production in adipose tissue. The relative mRNA levels (A) and the protein concentrations (B) of MCP-1 in epididymal adipose tissue were measured in *db/db* mice administered EP4 agonist (black bar) or vehicle (white bar). All values are mean ± SEM (A: n = 6 each, B: n = 6–8 each).
(TIF)

S2 Fig EP4 activation enhances the gene expression levels of PPARs in adipose tissue. Relative expression of *PPARδ*, *PPARγ*, and *PPARα* mRNA in epididymal adipose tissue from *db/db* mice administered EP4 agonist (black bar) or vehicle (white bar). All values are mean ± SEM (n = 4–5 each). ♯ p<0.01 vs. vehicle. PPAR, peroxidase proliferator–activated receptor.
(TIF)
