# Supplementary figures and images for: The Prostaglandin E2 Receptor EP4 Regulates Obesity-Related Inflammation and Insulin Sensitivity
Source: PLoS One. 2015 Aug 26;10(8):e0136304. doi: 10.1371/journal.pone.0136304 (PMC4550358; doi:10.1371/journal.pone.0136304)

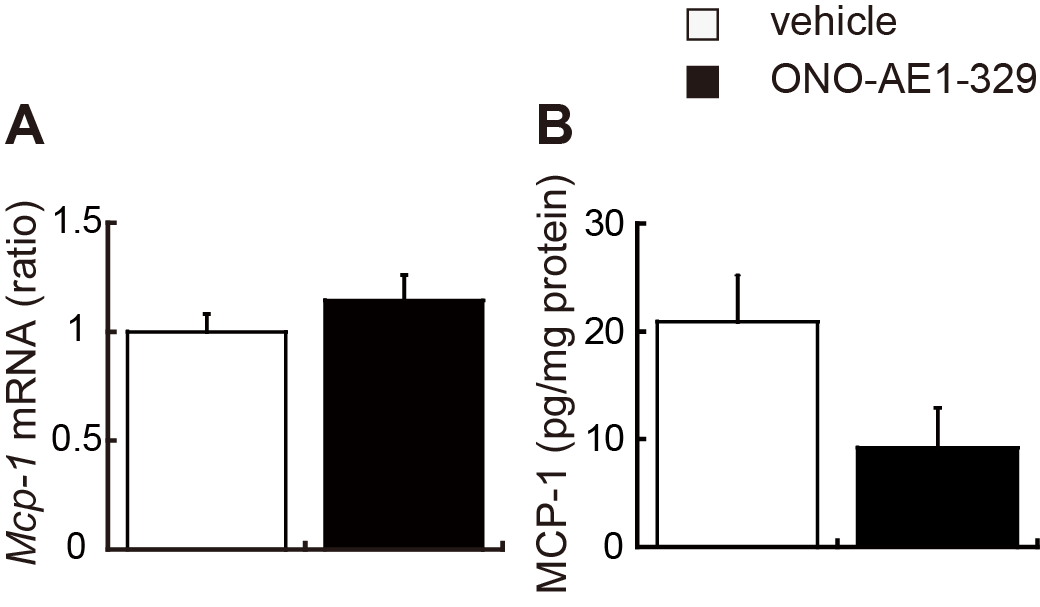

Supplement: S1 Fig — The relative mRNA levels (A) and the protein concentrations (B) of MCP-1 in epididymal adipose tissue were measured in db/db mice administered EP4 agonist (black bar) or vehicle (white bar). All values are mean ± SEM (A: n = 6 each, B: n = 6–8 each). (TIF) [file pone.0136304.s002.tif]

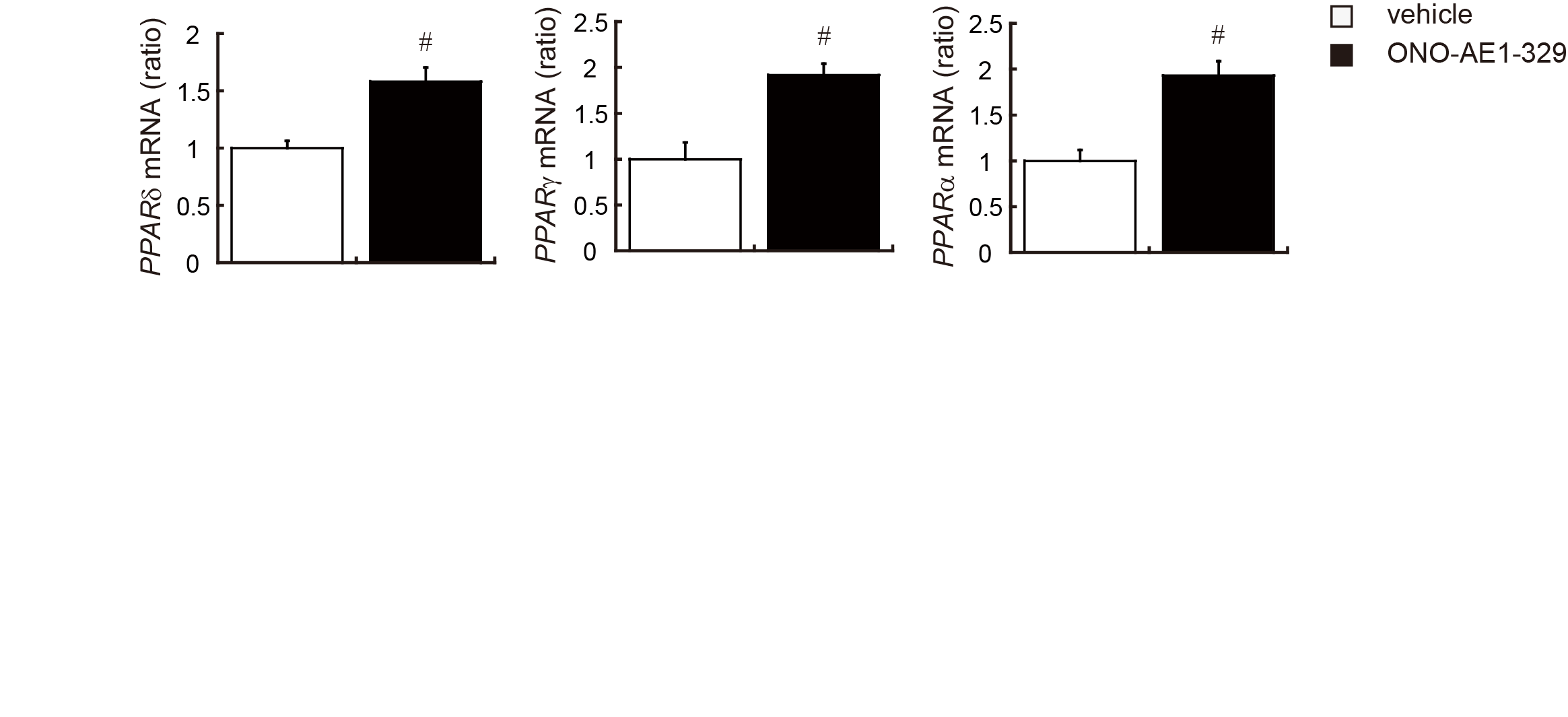

Supplement: S2 Fig — Relative expression of PPARδ, PPARγ, and PPARα mRNA in epididymal adipose tissue from db/db mice administered EP4 agonist (black bar) or vehicle (white bar). All values are mean ± SEM (n = 4–5 each). ♯ p<0.01 vs. vehicle. PPAR, peroxidase proliferator–activated receptor. (TIF) [file pone.0136304.s003.tif]
